# Gene Deletion in Barley Mediated by LTR-retrotransposon *BARE*

**DOI:** 10.1038/srep43766

**Published:** 2017-03-02

**Authors:** Yi Shang, Fei Yang, Alan H. Schulman, Jinghuan Zhu, Yong Jia, Junmei Wang, Xiao-Qi Zhang, Qiaojun Jia, Wei Hua, Jianming Yang, Chengdao Li

**Affiliations:** 1 National Barley Improvement Centre, Institute of Crop and Nuclear Technology Utilization, Zhejiang Academy of Agricultural Science, Hangzhou 310021, China; 2 Department of Genetics and Cell Biology, Yangtze University, Jingzhou, Hubei 434023, China; 3 Western Barley Genetics Alliance, Murdoch University, 90 South Street, Murdoch WA 6150, Australia; 4 Luke/BI Plant Genomics Lab, Institute of Biotechnology and Viikki Plant Science Centre, University of Helsinki, FIN-00014 Helsinki, Finland; 5 Green Technology, Natural Resources Institute Finland (Luke), Viikinkaari 1, FIN-00790 Helsinki, Finland; 6 College of Life Sciences, Zhejiang Sci-Tech University, Hangzhou 310018, China.

## Abstract

A poly-row branched spike (*prbs*) barley mutant was obtained from soaking a two-rowed barley inflorescence in a solution of maize genomic DNA. Positional cloning and sequencing demonstrated that the *prbs* mutant resulted from a 28 kb deletion including the inflorescence architecture gene *HvRA2*. Sequence annotation revealed that the *HvRA2* gene is flanked by two LTR (long terminal repeat) retrotransposons (*BARE*) sharing 89% sequence identity. A recombination between the integrase (IN) gene regions of the two *BARE* copies resulted in the formation of an intact *BARE* and loss of *HvRA2.* No maize DNA was detected in the recombination region although the flanking sequences of *HvRA2* gene showed over 73% of sequence identity with repetitive sequences on 10 maize chromosomes. It is still unknown whether the interaction of retrotransposons between barley and maize has resulted in the recombination observed in the present study.

The architecture of branched inflorescences in grasses depends on the developmental fate of primordia and axis orientation^1^. The rice (*Oryza sativa* L.) panicle generates several primary and secondary branches on which spikelets are produced. Sorghum and maize male inflorescences share a structure similar to that of rice. In barley (*Hordeum vulgare* L.) spikes, however, spikelets are borne directly on the main axis, the rachis, and there are no pedicels. A diagnostic feature of barley is the possession of three one-flowered spikelets at each rachis node^2^,^3^. Based on lateral spikelet size and fertility, barley is classified into two-rowed and six-rowed types. Two-rowed barley only has a central fertile spikelet with small and infertile lateral spikelets while the six-rowed barley has three fully-developed fertile spikelets.

The major genes that control row-type variation in barley are *Vrs1*^4^, *Int-c*^5^ and *Vrs4*^6^. The barley domestication gene *Vrs1*, located on the long arm of chromosome 2H, encodes a homeodomain-leucine zipper (HD-Zip) transcription factor that suppresses the development of lateral spikelets in two-rowed barley. Mutant *vrs1* results in a well-developed six-rowed phenotype^4^. *Int-c*, located on chromosome 4H, is an ortholog of the maize (*Zea mays.* L.) domestication gene, Teosinte branched 1 (*TB1*), a member of the TCP gene family encoding putative basic helix-loop-helix DNA-binding proteins^5^. The *Int-c* gene modifies lateral spikelet fertility in barley, and can influence the phenotypic effect of the *Vrs1* locus^7^.

*Vrs4* controls row-type and spikelet determinacy in barley; an induced mutation, *vrs4*, can convert the two-rowed to a six-rowed phenotype^5^,^8^. *Vrs4* is an ortholog of the maize inflorescence architecture gene RAMOSA2 (*RA2*), which encodes a transcriptional regulator that contains the lateral organ boundaries (LOB) domain. Expression analyses by mRNA *in situ* hybridization and microarray approaches showed that *Vrs4* is expressed very early during inflorescence development and controls the row-type pathway through *Vrs1* by negatively regulating the lateral spikelet fertility in barley. Moreover, the *Vrs4* gene is an important modifier of inflorescence development. Here, we report on a new mutant, poly-row and branched spike (*prbs*) obtained by soaking a two-rowed barley inflorescence in maize genomic DNA from a single cross hybrid^9^,^10^, and characterize its genetics, report its positional cloning, and analyze its origin.

## Results

### Mutant *prbs* resulted from deletion of the *Vrs4* gene

**Figure 1:**
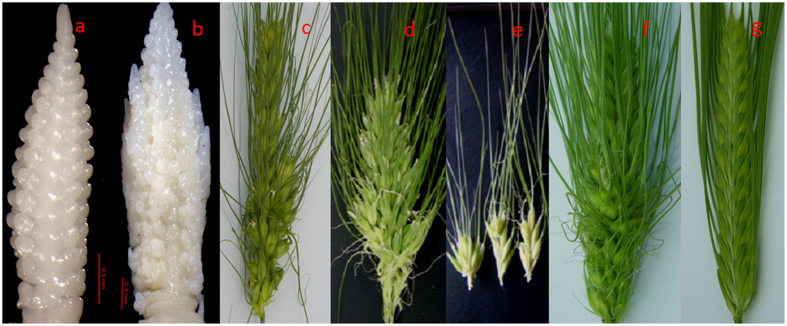
Morphology of developing and mature spikes. (**a**) normal spikes; (**b**,**c** and **d**) branching spikes; (**e**) branches of (**d**,**f**) mutant *prbs*; (**g**) wild parent Pudamai-2.

**Figure 2:**
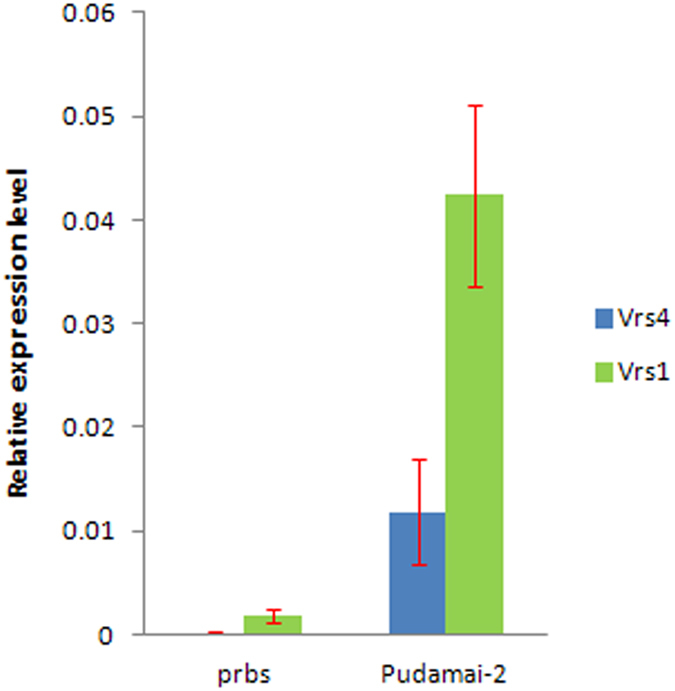
Relative expression level of *Vrs4* and *Vrs1* determined by quantitative RT-PCR in immature spikes at lemma primordium stage of the *prbs* mutant and wild parent Pudamai-2. Constitutively expressed *HvActin* was used for normalization.

The poly-row and branched spike (*prbs*) barley mutant was obtained by soaking a two-rowed barley inflorescence in maize genomic DNA solution^9^,^10^. The mutant *prbs* not only changes two-rowed barley into a poly-rowed form but also adds a spikelet row, forming irregular poly-row and branched spikes (Fig. 1). Genetic analysis indicated that the mutant phenotype was caused by a recessive gene, which has an epistatic effect on *Vrs1*^11^. The *prbs* was initially mapped to the centromere of the short arm of chromosome 3H^11^,^12^, a location similar to that of *vrs4.* Furthermore, the immature spikes of the *prbs* mutant under stereoscope are akin to the scanning electron microscopy images of the *Vrs4* immature spikes^12^. Three molecular markers (DQ327702, Cbic43, and Cbic44), closely linked with the *Vrs4* gene, co-segregated with the *prbs* gene in the *prbs*/Kunlun12 RIL and *prbs*/Zangqing 320 F_2_ populations (Supplementary Fig. 1). No fragment was amplified in the mutant plants using these three molecular markers. Similarly, three other primers covering the *Vrs4* gene sequence also failed to amplify a specific DNA fragment from either the *prbs* mutant or its progeny 11R258-95. Expression analyses revealed that the expression of *Vrs4* was not detected and the expression of *Vrs1* was significantly down-regulated in immature spikes at lemma primordium stage of the *prbs* mutant (Fig. 2). These results indicated that the *prbs* mutant may have resulted from a large deletion around the *Vrs4* gene.

### Identification of deletion region in *prbs* mutant

To identify the deletion region in the *prbs* mutant, a Morex BAC clone was identified that contains the *Vrs4* gene. PCR primers were designed at 2 kb intervals from 14 kb upstream to 22 kb downstream of the *Vrs4* gene and were tested on the *prbs* mutant, 11R258-95, Pudamai-2, and Morex. PCR primers located in the region from 3 kb upstream to 10 kb downstream of the *Vrs4* gene failed to amplify a specific DNA fragment in the *prbs* mutant and 11R258-95 but amplified a single band in Pudamai-2 and Morex instead. Sequencing revealed that amplicons represented a single product in Pudamai-2 and Morex. Primers designed from 3 to 13 kb upstream and 10 to 21 kb downstream of the *Vrs4* gene amplified a single band in all tested plants, but the amplicons represented multiple products when sequenced. These results did not support these regions arising from a single deletion event in the *prbs* mutant. However, an additional primer pair, Cbic123, matching a site 14 kb upstream of *Vrs4*, amplified a single band in all tested plants; the PCR product had 100% sequence identity among the *prbs* mutant, 11R258-95, Pudamai-2, and Morex. Another primer pair Cbic119, 22 kb downstream of *Vrs4*, also amplified a single band in all tested plants (sequencing was identical in all tested lines). These results revealed that the deletion sequence was from ~13 to 36 kb in the *prbs* mutant.

**Figure 3:**
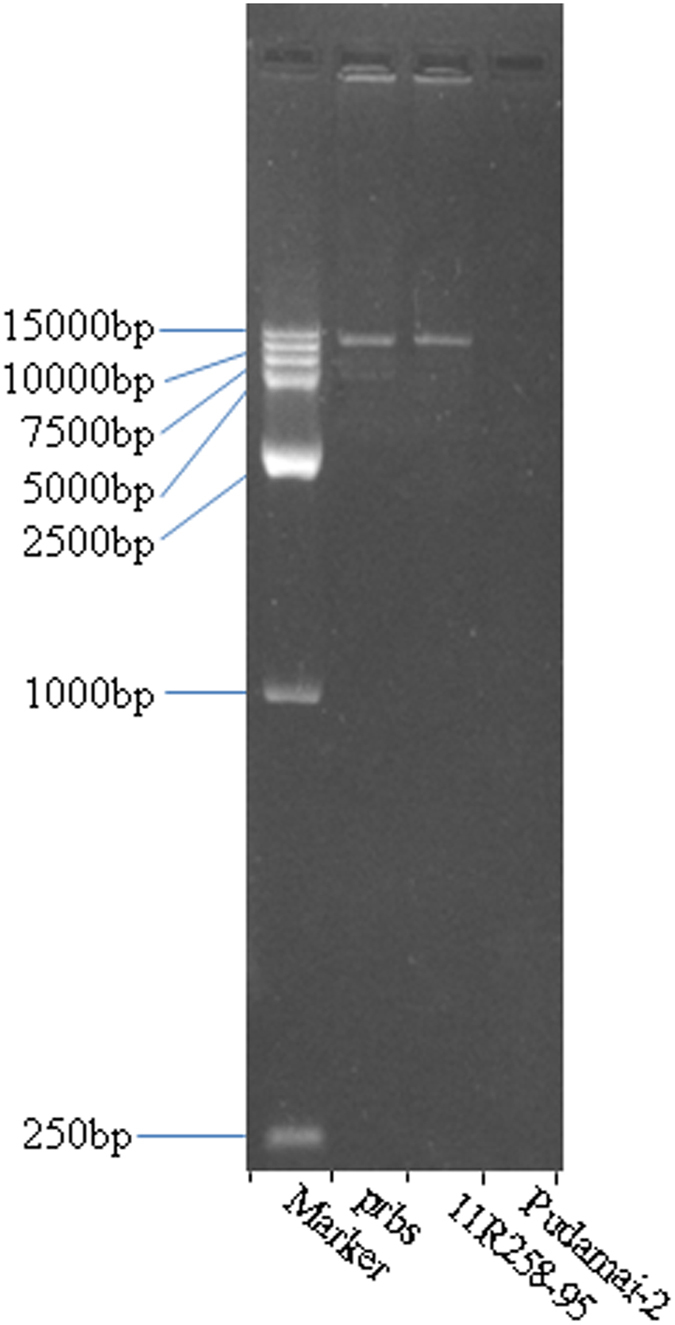
PCR-amplification of the 15-kb recombination sequence fragment in the *prbs* mutant and RIL 11R258-95.

After failure to amplify a single DNA fragment using many PCR primers in the target region, long-range PCR was used to isolate the sequence covering the *prbs* mutation. Based on the above PCR test results, PCR primers Cbic131 and Cbic132 were designed for this purpose; the forward primer was near the site of primer Cbic123 and the reverse primer near the site of primer Cbic119, as both have been confirmed to amplify a single copy of DNA from the control varieties and mutants. A 15 kb fragment was successfully amplified from both the *prbs* mutant and 11R258-95 (Fig. 3), whereas the control PCRs using DNA from Pudamai-2 and Morex as a template failed to amplify. The amplification product from *prbs* was 14,715 bp (accession number KU758926).

**Figure 4:**
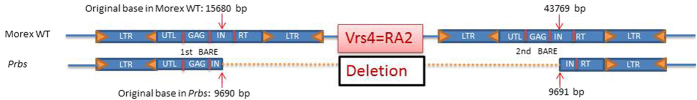
Schematic comparison the Morex and *prbs* mutant sequences. The *Vrs4* gene is flanked by two BARE retrotransposons which are bounded by 1.8 kb long terminal repeats (LTRs) and contains coding domains of GAG, integrase (IN), reverse transcriptase (RT), and RNaseH in Morex. The two BAREs sequences are highly conserved and share 89% of sequence identity. The *prbs* mutant has 28 kb deletion including the entire *Vrs4* gene and parts of the two retrotransposons. The non-retrotransposon sequence in the wild parent Pudamai 2 share 100% of sequence identity with Morex.

We identified a 48,951 bp sequence from Morex using the 14,715 bp *prbs* sequence as query in BLASTN searches of the Morex genome database (http://webblast.ipk-gatersleben.de/barley/viroblast.php). Alignment of these two sequences showed that a 27,804 bp sequence in Morex, extending from nt 15,680 to nt 43,769 and containing the entire *Vrs4* gene, is deleted in *prbs* (Fig. 4). Sequence analysis demonstrated that the *Vrs4* gene in Morex is flanked by two long terminal repeat (LTR) retrotransposons located, respectively, at nt 10,967 to 19,828 upstream and nt 38,791 to nt 47,684 downstream (Fig. 4). A search of the Triticeae Repetitive Elements (TREP) database revealed high similarity to the retrotransposon RLC *BARE1* B consensus-1 (TREPACC = TREP3133) with 90% and 98% of sequence identity, respectively, for the two elements. We refer to the retrotransposon upstream of *Vrs4* as *BARE up*, and the one downstream of *Vrs4* as *BARE down*. The two share 90% identity, are bound by 1.8 kb LTRs, and contain the full-length open reading frame encoding Gag, aspartic proteinase (AP), integrase (IN), reverse transcriptase (RT), and RNaseH (RH) expected for canonical *BARE1* elements^13^ (Fig. 4).

We further sequenced part of the deletion from the wild parent Pudamai-2, which contains a complete *Vrs4* gene. A phylogenetic tree was constructed using the MEGA6 program^14^ with the minimum evolution method. The results showed that the *Vrs4* haplotype in Pudamai-2 was similar to haplotype 8 (Supplementary Fig. 2) which was characterized by an insertion of TA bases in the 5′ UTR of the gene^6^ and was mainly distributed in Asia. Thus, the *prbs* mutant resulted from a deletion of the entire functional gene *HvRA2*, a barley ortholog of the maize inflorescence architecture gene RAMOSA2 (*RA2*), which thereby transformed a two-row barley into a poly-row branched structure.

### Recombination between the integrase genes of two *BARE*s formed *prbs*

**Figure 5:**
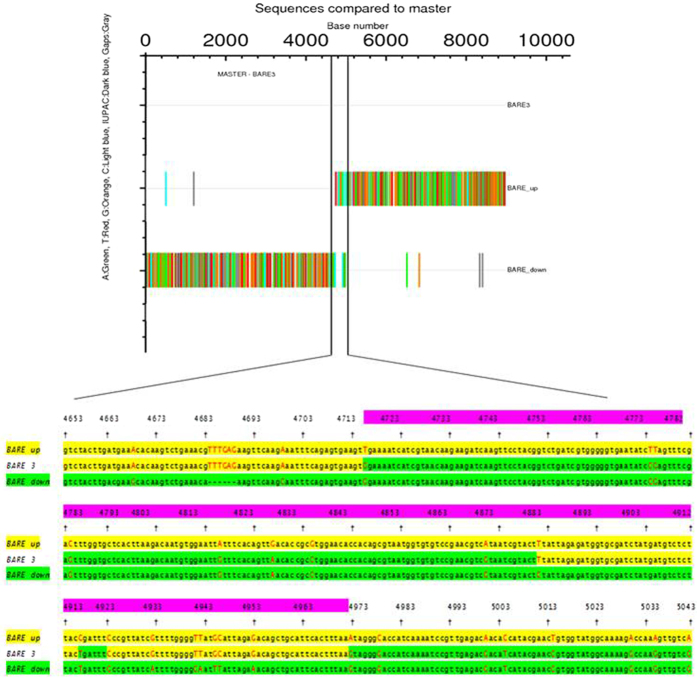
Alignment of the three LTR retrotransposons: *BARE up, BARE down, BARE 3*, shows recombination region. Red capital letters marked Indel sites and black lower letters marked consensus sequences. Yellow background show *BARE up* sequences and their homologous sequences in *BARE 3.* Green background show *BARE down* sequences and their homologous sequences in *BARE 3.* Purple red show the recombination region decided by the alignment.

Sequence analysis of the 15 kb region from *prbs* identified a single *BARE* element of the canonical 8.9 kb in length. Alignment revealed that the 5′ part of the *prbs* (nt 1 to 4,714) *BARE* was identical to *BARE up*, whereas the 3′ part (4,972 to 8,918 bp) was the same as *BARE down* (Fig. 5). The joint between the two halves is between nt 4,715 and nt 4,971 in the *prbs BARE* (Fig. 5), within the integrase (IN) domain, corresponding to position nt 9,402–9,690 in the cloned *prbs* fragment (accession number KU758926). Retrotransposon integration generates a direct-repeat target-site duplication (TSD) flanking the individual element, as a consequence of repair of the staggered cut made by the integrase^15^. *BARE up* is flanked by imperfect CCAAG TSDs and *BARE down* by a perfect pair of CTGAA motifs. The *BARE* in *prbs* is flanked by CCAAG and CTGAA, supporting the origin of this *BARE* by recombination. Moreover, the upstream and downstream sequences surrounding the single *BARE* in *prbs* correspond, respectively, to that upstream of *BARE up* and downstream of *BARE down*. Thus, the *HvRA2* gene, flanked by two *BARE* elements in Pudamai-2, was deleted by recombination between them, thereby generating the *prbs* mutation.

### Search the maize genome for sequence similarity

To investigate the possible role of maize DNA in forming the *prbs* mutation, we used the region spanning the recombination zone in the *BARE* elements (9,402–9,690 bp) to carry out a BLASTn search the MaizeGDB B73 reference genome sequence. No maize-specific sequence was found in the region. Hence, it appears that no maize DNA has been inserted into the *prbs* region and that the *prbs* phenotype results solely from the *Vrs4* gene deletion.

As an alternative to insertion, the maize DNA may have played a role through sequence similarity at the recombination point. The region of recombination in *prbs* corresponds to the most conserved part of the integrase gene, which is the core domain that includes the D-D-35-E active site motif^16^. The recombination itself took place in the region between the second Asp and the Glu of the active site; a BLASTn search of this region against the MaizeGDB B73 reference genome sequence found more than 120 matches between 73% and 80% identity, containing multiple stretches of ~10 nt perfect identity, dispersed over all maize chromosomes (Table 1). Given their numbers, it is highly likely that these BLAST matches correspond to members of the *Copia* superfamily in the maize genome, which comprises ~425–485 Mb of the maize genome^17^, the universal presence of integrase in intact, autonomous LTR retrotransposons. Further research is required whether foreign DNA may induce recombination through sequence similarity, especially when the foreign DNA exist in high concentration or high copy numbers.

## Discussion

Barley is classified as two-rowed or six-rowed based on lateral spikelet size and fertility. Two-rowed barley has a central fertile spikelet and two infertile lateral spikelets, and six-rowed barley has three fully-developed fertile spikelets. *Vrs4* is an ortholog of the maize (*Zea mays.* L.) inflorescence architecture gene *RAMOSA2 (RA2*), which encodes a LOB-domain-containing transcriptional regulator^18^,^19^,^20^. *Vrs4* controls row-type variation and modifies inflorescence development in barley (*Hordeum vulgare.* L)^6^. Expression analyses of mRNAs by *in situ* hybridization and microarray analysis revealed that *Vrs4* is expressed very early during inflorescence development and controls the row-type pathway in barley through *Vrs1*, a negative regulator of lateral spikelet fertility.

The *prbs* mutant was obtained from Pudamai-2, which has the normal *Vrs4* gene and a two-rowed phenotype, by soaking the barley inflorescence in maize genomic DNA solution. In the mutant, *Vrs4* is deleted through a recombination between two *BARE* retrotransposons on either side of the *Vrs4* gene. LTR retrotransposons are known to recombine; the recombination between two LTRs of a single element, which results in deletion of the internal domain of the retrotransposon and generates a solo LTR, has been studied^16^. The process of retrotransposon replication generates LTRs that are identical at the moment of integration^21^; the accumulation of mutations in the LTRs at the neutral rate after that allows for the estimation of the age of the integrated element^22^. Genome-wide analyses show that the average half-life of a retrotransposon in the *Copia* superfamily, which includes *BARE*, is 859,000 years, or a rate of 1.16 × 10^−6^ events per element per generation, in the grass *Brachypodium distachyon*, which loses retrotransposons through recombination relatively rapidly^23^. In barley and other plants, the rate of solo LTR formation varies considerably between retrotransposon families and also between chromosomes and regions^20^,^23^,^24^. Analyses of the frequency of recombination events between internal retrotransposon domains, such as the generated *prbs* reported here, have not been made and are difficult to identify in the absence of novel phenotypes.

Recombination between the LTRs of two different elements can generate a concatenated structure comprising two internal domains flanking a single, recombinant LTR, which results in the loss of the intervening genomic sequence, including any gene that happens to be there. A quantitative PCR survey of the barley genome for such structures with three LTRs and two internal domains showed that their presence in about 4.3 × 10^3^ copies per haploid genome^23^. While this indicates the potential for gene loss through recombination of retrotransposons flanking a gene, especially given that the gene islands^25^ are flanked by retrotransposon “seas” which increases the intervening distance between recombining retrotransposon sequences and appears to be correlated with decreasing recombination frequency^20^.

The question arises as to whether the maize DNA soaking procedure is connected to the recombination that generated the *prbs* mutant. Our procedure and the other methods introduce foreign DNA into the megagametophyte before fertilization^26^,^27^. Whether or not any foreign DNA is integrated, the presence of extra chromosomal or cytoplasmic DNA triggers a range of defense responses in animals^28^,^29^, mediated by DNA recognition by proteins including STING (also called MITA, MPYS, TMEM173, or ERIS)^30^, specific toll-like receptors (TLRs)^31^, Z-DNA binding proteins (ZBP-1, DLM-1, or DAI)^32^, and Mre11 (meiotic recombination 11)^33^. Mre11 is particularly intriguing because, together with RAD50 and NBS1, is a part of the MRN complex and has been shown to play a vital role in double-strand break (DSB) repair^34^ in plants, which is an intermediate step in recombination.

The maize genome contains 404,000 *Copia* superfamily retrotransposons^35^,^36^; the integrase domains of these are very similar to the integrase core domain of *BARE* that underwent recombination in *prbs.* We speculate whether the homologous maize and barley integrase sequences may have interacted with each other, mediating the recombination. Expression analyses, *in situ* hybridization and microarrays revealed that *Vrs4* is actively expressed during inflorescence development^6^, corresponding to the stage at which the barley inflorescence was soaked in maize genomic DNA to generate the *prbs* mutant. Due to its transcriptional activity, this region is likely to have an open chromatin conformation, which could provide an opportunity for maize DNA to interact at the *BARE* integrase domains and promote the recombination. The high concentration of conserved retrotransposon sequences would make binding and recombination in a retrotransposon sequence more likely than elsewhere. While recombinations between endogenous retroviruses (ERVs)—which are structurally identical to LTR retrotransposons—have caused genic deletions through recombination^37^, to our knowledge, there has not been an earlier demonstration of this in plants.

Horizontal gene transfer (HGT) is well documented in prokaryotic genome evolution. It is relatively clear that there are several HGT pathways, including transformation, conjugation, and transduction. In eukaryotes, direct DNA exchanges may occur during grafting^38^, symbiosis^39^,^40^, parasitism^41^, pathogenesis^42^, and epiphyte or entophyte^43^. Some vectors, such as pollen^43^, fungi^44^, bacteria^45^, viruses^46^, plasmids^38^, insects^47^ and transposons^43^, may also be involved in HGT. Transposable elements (TEs) have been recognized as important vectors for the horizontal movement of genes between eukaryotic genomes^48^,^49^,^50^,^51^. Transposons, with their inherent ability to mobilize, can proliferate and integrate into genomic DNA and generate HGTs with ease^52^. Transposons have also captured and transduced genomic DNA sequences in both *Daphnia pulex*^53^ and *Drosophila* species^52^. The transfer of Mu-like transposons between *Setaria* and rice has been documented^48^,^54^. LTR retrotransposons can produce virus-like particles, which may work as more frequent vectors for HGT^52^,^55^. Such cases have been demonstrated in LTR-retrotransposon *RIRE1* within the genus *Oryza*^56^ and the LTR-retrotransposon Route66 in *Poaceae*^50^. With the increasing availability of eukaryotic genome sequences, more evidence will be available that plants are also likely to undergo HGT. However, the results have been based on incongruences in molecular phylogenetic trees. On the other hand, there are numerous reports in the literature that have directly introduced foreign DNA by injecting exogenous DNA or directly DNA soaking or pollen tube pathway into rice, barley, wheat, sorghum, maize, cotton, oats, rye, cucumber, pumpkin, kidney bean and soybean to create new genetic variations (Supplementary Table 7). RAPD, AFLP and SSR molecular markers have been used to test DNA transfers between species in several studies^57^,^58^,^59^. However, no study has demonstrated how the exogenous DNA causes genetic variation in other species. Our study provides preliminary evidence that LTR-retrotransposon-mediated gene deletion/insertion may play a role in direct gene transfer between different species.

In addition to act as potential vectors for horizontal gene transfer, transposable elements are also responsive and susceptible to environmental changes. It is well documented that stresses could activate TE to generate new genetic diversity^60^. This is especially true for LTR-retrotransposons as the LTR is sufficient in itself to activate TE transcription in response to stress. It is possible that soaking the barley spike in the maize DNA solution created stress conditions for the developing spikelets, which activated LTR-TE mediated recombination. In this scenario, the maize genomic DNA may be not essential for the mutation. Further research is required to test this assumption by soaking the developing barley spikes in water or salt solution to provide similar stresses for identification of new mutants.

## Materials and Methods

### Plant materials

A poly-row branched spike (*prbs*) barley mutant was obtained by soaking a two-rowed barley inflorescence (cv. Pudamai-2) in maize genomic DNA solution^61^. The method followed that described earlier for wheat^62^. Flowering barley spikes were soaked in total maize DNA at 1.6 ug/ul in 0.1 × SSC for 24 hours. After soaking, the head was moved from the solution and air-dried under ambient conditions. Plants were self-pollinated and seeds harvested. The mutant was identified at flowering of the next generation plants.

Genetic mapping was conducted in two populations: one recombinant inbred line (RIL, F_2:6_) population consisting of 207 plants derived from a cross between the *prbs* mutant and a six-rowed barley cultivar Kunlun 12, and an F_2_ population consisting of 285 spike mutant plants derived from a cross between the *prbs* mutant and a six-rowed barley cultivar Zangqing 320. The *prbs* mutant, RIL 11R258-95 with a branched spike phenotype, Pudamai-2, and var. Morex were used for DNA sequence analysis.

### Genomic DNA extraction and genotype analysis

Genomic DNA was extracted from leaves of individual plants and their parents using a modified CTAB method^63^. DNA samples were quantified using a Unican UV300 UV/Vis spectrometer (Thermo Electron Corporation, Cambridge, UK), and then adjusted to 25 ng/μl. Because a DQ327702 marker associated with the mutant is closely linked with the *Vrs4* gene^6^, new molecular markers Cbic43 and Cbic44 were designed around the *Vrs4* gene using the barley genome sequence^14^ from the IPK Barley BLAST server (http://webblast.ipk-gatersleben.de/barley/viroblast.php). Primer pairs specific to the *Vrs4* gene (AS12, AS34, and AS56)^6^ were designed for *Vrs4* haplotype analysis. Primers were synthesized by Shanghai Sunny Biotechnology (Shanghai, China). PCR reactions were performed in 10 μL volumes containing approximately 25 ng genomic DNA, 0.2 μM of each primer, and 5 μL 2 × Taq Master Mix (Gene Solution, Shanghai, China) using the following program: 94 °C for 3 min, 32 cycles of 94 °C for 30 sec, 55 °C for 45 sec, 72 °C for 1 min, and 72 °C for 5 min. PCR products were separated on 8% polyacrylamide gels.

### Cloning of the deletion in mutant *prbs*

BAC sequences were identified by blasting the *Vrs4* against the International Barley Genome Sequencing Consortium database (unpublished data). PCR primer pairs were designed at 2 kb intervals in the region near the *Vrs4* locus using the Primer-Blast tool (http://www.ncbi.nlm.nih.gov/tools/primer-blast/). PCR reactions were described as above. Annealing temperatures were optimized for each primer pair (Supplementary Table 1). PCR products were examined by electrophoresis on 1% agarose gels. Long-range PCRs were performed in 50 μL reactions containing 1 × buffer, 5 μL template DNA, 0.4 μM of each primer, 400 μM each deoxyribonucleotide, and 2.5 U LA Taq DNA polymerase (Takara, Dalian, China) using the following program: 94 °C for 3 min, 32 cycles of 98 °C for 10 sec, 68 °C for 15 min, and 68 °C for 20 min. PCR products were examined by electrophoresis using a 0.8% agarose gel, analyzed by Bio-Red Quantity One gel image analysis system and sequenced by Shanghai RuiDi Biological Technology (Shanghai, China).

### Quantitative RT-PCR

RNA was extracted from immature spikes at lemma primordium stage of the *prbs* mutant and wild parent Pudamai-2 using Spin Column Plant total RNA Purification Kit (Sanggon Biotech (Shanghai) Co.,Ltd). cDNA was prepared from 1 ug RNA using AMV First Strand cDNA Synthesis Kit (Sanggon Biotech (Shanghai) Co., Ltd). qPCR reactions were performed using SYBR Green (SG Fast qPCR Master Mix (HighRox), BBI) and the Applied Biosystems Stepone plus Real-time PCR System. The Real-time PCR assays were performed in triplicate for each cDNA sample. *Vrs4*^6^, *Vrs1*^4^ and *HvActin*^6^ primer sequences used for quantitative RT-PCR. The *HvActin* gene was used as reference gene for normalization.

### Sequence analysis

Alignments of mutant *prbs* and barley genomic sequences were constructed using MEGA 6.0^14^ and BLASTN 2.3.0+ 64. The prediction of transposable elements was identified through LTR Finder 1.05 (http://tlife.fudan.edu.cn/ltr_finder/)^65^ and BLAST^66^ against the Triticeae Repetitive Element (TREP) database (http://wheat.pw.usda.gov/GG2/blast.shtml). Searches for sequence homology to maize was conducted with MaizeGDB against the sequence database B73 RefGen_v3 (MGSC), using the BLAST program BLASTN^63^ with an E-value cutoff <1e-50.

## Additional Information

**How to cite this article:** Shang, Y. *et al*. Gene Deletion in Barley Mediated by LTR-retrotransposon *BARE. Sci. Rep.* **7**, 43766; doi: 10.1038/srep43766 (2017).

**Publisher's note:** Springer Nature remains neutral with regard to jurisdictional claims in published maps and institutional affiliations.

## Supplementary Material

Supplementary Information

## Figures and Tables

**Table 1:** Sequence alignment of the Morex deletion with the maize genome sequence.

	Morex-start	Morex-end	Length	Highest percent identity	Highest e value	Hit number	Detail alignment results
1	15345	16193	848	73.01	1.484e-75	926	Table S2, Fig. S3
	43155	43887	732				
2	16423	18000	1577	72.61	2.035e-121	1019	Table S3, Fig. S4
	44227	45804	1577				
3	18046	18087	41	97.62	3.38E-12	879	Table S4, Fig. S5
	45850	45891	41				
4	27390	27543	153	81.1	5.543e-169	35	Table S5, Fig. S6
5	25868	26648	780	88.2	3.106e-45	1	Table S6, Fig. S7

## References

[b1] Doust A. N. & Kellogg E. A. Inflorescence diversification in the panicoid “bristle grass” clade (Paniceae, Poaceae): evidence from molecular phylogenies and developmental morphology. Am. J. Bot. 89, 1203–1222 (2002). 21665721 10.3732/ajb.89.8.1203

[b2] Sreenivasulu N. & Schnurbusch T. A genetic playground for enhancing grain number in cereals. Trends Plant Sci. 17, 91–101 (2012). 22197176 10.1016/j.tplants.2011.11.003

[b3] Forster B. P. . The barley phytomer. Ann. Bot. 100, 725–733 (2007). 17901062 10.1093/aob/mcm183 PMC2749621

[b4] Komatsuda T. . Six-rowed barley originated from a mutation in a homeodomain-leucine zipper I-class homeobox gene. Proc. Natl. Acad. Sci. USA 104, 1424–1429 (2007). 17220272 10.1073/pnas.0608580104 PMC1783110

[b5] Ramsay L. . INTERMEDIUM-C, a modifier of lateral spikelet fertility in barley, is an ortholog of the maize domestication gene TEOSINTE BRANCHED 1. Nat. Genet. 43, 169–172 (2011). 21217754 10.1038/ng.745

[b6] Koppolu R. . Six-rowed spike4 (Vrs4) controls spikelet determinacy and row-type in barley. Proc. Natl. Acad. Sci. USA 110, 13198–13203 (2013). 23878219 10.1073/pnas.1221950110 PMC3740847

[b7] Lundqvist U. & Lundqvist A. Induced intermedium mutants in barley: origin, morphology and inheritance. Hereditas. 108, 13–26 (1988).

[b8] Lundqvist U., Franckowiak J. D. & Konishi T. New and revised descriptions of barley genes. Barley Genet. Newsl. 26, 22–516 (1997).

[b9] Liu S. . Studies on inheritance and spike characters of poly-row-and-branched spike mutant in barley. Fujian Science and Technology of Rice and Wheat. 18, 37–39 (2000).

[b10] Ji H., Chen Q. & Lin X. Study on poly-row barley with multi-branches of spikelets emerged by directly of DNAs. Journal of Fujian Agriculture University 24, 9–13 (1995).

[b11] Huang B., Wu W., Liu S. & Huang Z. Genetic Analysis on Poly-row-and-branched Spike Mutant in Barley. Hereditas (Beijing) 26, 903–906 (2004). 15640124

[b12] Shang Y. . Characterization and mapping of a Prbs gene controlling spike development in Hordeum vulgare L. Genes Genomics 36, 275–282 (2014).

[b13] Suoniemi A., Tanskanen J., Pentikainen O., Johnson M. S. & Schulman A. H. The core domain of retrotransposon integrase in Hordeum: predicted structure and evolution. Mol. Biol. Evol. 15, 1135–1144 (1998). 9729878 10.1093/oxfordjournals.molbev.a026021

[b14] Tamura K., Stecher G., Peterson D., Filipski A. & Kumar S. MEGA6: Molecular Evolutionary Genetics Analysis version 6.0. Mol. Biol. Evol. 30, 2725–2729 (2013). 24132122 10.1093/molbev/mst197 PMC3840312

[b15] Krishnan L. & Engelman A. Retroviral integrase proteins and HIV-1 DNA integration. J. Biol. Chem. 287, 40858–40866 (2012). 23043109 10.1074/jbc.R112.397760 PMC3510789

[b16] Vitte C. & Panaud O. Formation of solo-LTRs through unequal homologous recombination counterbalances amplifications of LTR retrotransposons in rice Oryza sativa L. Mol. Biol. Evol. 20, 528–540 (2003). 12654934 10.1093/molbev/msg055

[b17] Estep M. C., DeBarry J. D. & Bennetzen J. L. The dynamics of LTR retrotransposon accumulation across 25 million years of panicoid grass evolution. Heredity (Edinb). 110, 194–204 (2013). 23321774 10.1038/hdy.2012.99 PMC3554455

[b18] Bortiri E. . Ramosa2 Encodes a LATERAL ORGAN BOUNDARY Domain Protein That Determines the Fate of Stem Cells in Branch Meristems of Maize. Plant Cell 18, 574–585 (2006). 16399802 10.1105/tpc.105.039032 PMC1383634

[b19] Shirasu K., Schulman A. H., Lahaye T. & Schulze-Lefert P. A contiguous 66-kb barley DNA sequence provides evidence for reversible genome expansion. Genome Res. 10, 908–915 (2000). 10899140 10.1101/gr.10.7.908 PMC310930

[b20] Baidouri M. E. & Panaud O. Comparative Genomic Paleontology Across Plant Kingdom Reveals The Dynamics Of TE-driven Genome Evolution. Genome Biol Evol. 5, 954–965 (2013). 23426643 10.1093/gbe/evt025 PMC3673626

[b21] Schulman A. H. Retrotransposon replication in plants. Curr Opin Virol. 3, 604–614 (2013). 24035277 10.1016/j.coviro.2013.08.009

[b22] SanMiguel P., Gaut B. S., Tikhonov A., Nakajima Y. & Bennetzen J. L. The paleontology of intergene retrotransposons of maize. Nat. Genet. 20, 43–45 (1998). 9731528 10.1038/1695

[b23] Vogel J. P. . Genome sequencing and analysis of the model grass Brachypodium distachyon. Nature. 463, 763–768 (2010). 20148030 10.1038/nature08747

[b24] Mayer K. F. . A physical, genetic and functional sequence assembly of the barley genome. Nature 491, 711–716 (2012). 23075845 10.1038/nature11543

[b25] Liu R. . A GeneTrek analysis of the maize genome. Proc. Natl. Acad. Sci. USA 104, 11844–11849 (2007). 17615239 10.1073/pnas.0704258104 PMC1913904

[b26] Zhou G. Y. . Introduction of exogenous DNA into cotton embryos. Meth. Enzymol. 101, 433–481 (1983). 6577258 10.1016/0076-6879(83)01032-0

[b27] Peña A. D. L., Lörz H. & Schell J. Transgenic rye plants obtained by injecting DNA into young floral tillers. Nature. 325, 274–276 (1987).

[b28] Barber G. N. STING-dependent cytosolic DNA sensing pathways. Trends Immunol. 35, 88–93 (2014). 24309426 10.1016/j.it.2013.10.010

[b29] Ma Z. . Modulation of the cGAS-STING DNA sensing pathway by gammaherpesviruses. Proc. Natl. Acad. Sci. USA 112, 4306–4315 (2015). 10.1073/pnas.1503831112 PMC4534226 26199418

[b30] Abe T. . STING Recognition of Cytoplasmic DNA Instigates Cellular Defense. Mol. Cell. 50, 5–15 (2013). 23478444 10.1016/j.molcel.2013.01.039 PMC3881179

[b31] Kawai T. & Akira S. Toll-like Receptors and Their Crosstalk with Other Innate Receptors in Infection and Immunity. Immunity 34, 637–650 (2011). 21616434 10.1016/j.immuni.2011.05.006

[b32] Takaoka A. . DAI (DLM-1/ZBP1) is a cytosolic DNA sensor and an activator of innate immune response. Nature 448, 501–505 (2007). 17618271 10.1038/nature06013

[b33] Kondo T. . DNA damage sensor MRE11 recognizes cytosolic double-stranded DNA and induces type I interferon by regulating STING trafficking. Proc. Natl. Acad. Sci. USA 110, 2969–2974 (2013). 23388631 10.1073/pnas.1222694110 PMC3581880

[b34] Samanic I., Cvitanic R., Simunic J. & Puizina J. Arabidopsis thaliana MRE11 is essential for activation of the cell cycle arrest, transcriptional regulation and the DNA repair upon the induction of double- stranded DNA breaks. Plant Biol (Stuttg). 18, 681–694 (2016). 27007017 10.1111/plb.12453

[b35] Tenaillon M. I., Hufford M. B., Gaut B. S. & Rossibarra J. Genome size and transposable element content as determined by high-throughput sequencing in maize and Zea luxurians. Genome Biol Evol. 3, 219–229 (2011). 21296765 10.1093/gbe/evr008 PMC3068001

[b36] Schnable P. S. . The B73 maize genome: complexity, diversity, and dynamics. Science. 326, 1112–1115 (2009). 19965430 10.1126/science.1178534

[b37] Shuvarikov A. . Recurrent HERV-H-mediated 3q13.2-q13.31 deletions cause a syndrome of hypotonia and motor, language, and cognitive delays. Hum. Mutat. 34, 1415–1423 (2013). 23878096 10.1002/humu.22384 PMC4599348

[b38] Stegemann S. & Bock R. Exchange of genetic material between cells in plant tissue grafts. Science 324, 649–651 (2009). 19407205 10.1126/science.1170397

[b39] Finan T. M. Evolving insights: symbiosis islands and horizontal gene transfer. J. Bacteriol. 184, 2855–2856 (2002). 12003923 10.1128/JB.184.11.2855-2856.2002 PMC135049

[b40] Burger G. & Lang B. F. Parallels in genome evolution in mitochondria and bacterial symbionts. IUBMB Life 55, 205–212 (2003). 12880200 10.1080/1521654031000137380

[b41] Yoshida S., Maruyama S., Nozaki H. & Shirasu K. Horizontal gene transfer by the parasitic plant Striga hermonthica. Science 328, 1128–1128 (2010). 20508124 10.1126/science.1187145

[b42] Sharp A. J. . Discovery of previously unidentified genomic disorders from the duplication architecture of the human genome. Nat. Genet. 38, 1038–1042 (2006). 16906162 10.1038/ng1862

[b43] Bock R. The give-and-take of DNA: horizontal gene transfer in plants. Trends Plant Sci. 15, 11–22 (2010). 19910236 10.1016/j.tplants.2009.10.001

[b44] Richards T. A. . Phylogenomic analysis demonstrates a pattern of rare and ancient horizontal gene transfer between plants and fungi. Plant Cell. 21, 1897–1911 (2009). 19584142 10.1105/tpc.109.065805 PMC2729602

[b45] Broothaerts W. . Gene transfer to plants by diverse species of bacteria. Nature 433, 629–633 (2005). 15703747 10.1038/nature03309

[b46] Hull R., Harper G. & Lockhart B. Viral sequences integrated into plant genomes. Annu Rev Phytopathol. 5, 362–365 (2000). 10.1016/s1360-1385(00)01723-4 11203277

[b47] Dieterich C. . The Pristionchus pacificus genome provides a unique perspective on nematode lifestyle and parasitism. Nat. Genet. 40, 1193–1198 (2008). 18806794 10.1038/ng.227 PMC3816844

[b48] Diao Y. . Next-generation sequencing reveals recent horizontal transfer of a DNA transposon between divergent mosquitoes. PloS One 6, e16743 (2011). 21379317 10.1371/journal.pone.0016743 PMC3037385

[b49] Novick P., Smith J., Ray D. & Boissinot S. Independent and parallel lateral transfer of DNA transposons in tetrapod genomes. Gene. 449, 85–94 (2010). 19747963 10.1016/j.gene.2009.08.017

[b50] Roulin A. . Whole genome surveys of rice, maize and sorghum reveal multiple horizontal transfers of the LTR-retrotransposon Route66 in Poaceae. BMC Evol. Biol. 9, 58 (2009). 19291296 10.1186/1471-2148-9-58 PMC2664808

[b51] Sormacheva I. . Vertical evolution and horizontal transfer of CR1 non-LTR retrotransposons and Tc1/mariner DNA transposons in Lepidoptera species. Mol. Biol. Evol. 9, 3685–3702 (2012). 10.1093/molbev/mss181 22826456

[b52] Loreto E. L. S., Carareto C. M. A. & Capy P. Revisiting horizontal transfer of transposable elements in Drosophila. Heredity. 100, 545–554 (2008). 18431403 10.1038/sj.hdy.6801094

[b53] Schaack S., Choi E., Lynch M. & Pritham E. J. DNA transposons and the role of recombination in mutation accumulation in Daphnia pulex. Genome Biol. 11, R46 (2010). 20433697 10.1186/gb-2010-11-4-r46 PMC2884549

[b54] Diao X., Freeling M. & Lisch D. Horizontal transfer of a plant transposon. PLoS Biol. 4, 119 (2006). 10.1371/journal.pbio.0040005 PMC1310652 16336045

[b55] Silva J. C., Loreto E. L. & Clark J. B. Factors that affect the horizontal transfer of transposable elements. Curr Issues Mol Biol. 6, 57–71 (2004). 14632259

[b56] Roulin A., Piegu B., Wing R. A. & Panaud O. Evidence of multiple horizontal transfers of the long terminal repeat retrotransposon RIRE1 within the genus Oryza. Plant J. 53, 950–959 (2008). 18088314 10.1111/j.1365-313X.2007.03388.x

[b57] Luo H., Zhong B., Yang Z., Li Y. & He G. The SSR molecular evidence of rice transformation via pollen tube pathway. Mol. Plant Breed. 2, 501–505 (2004).

[b58] Liu P. & Kang Z. Oat (Avena sat iva L) exogenous DNA introduction into common wheat and PAPD analysis. Agricultural Research in the Arid Areas. 24, 100–103 (2006).

[b59] Wang S. . Molecular verification of DNA flow from wild rice (O. minuta) to cultivated rice. Scientia Agricultura Sinica. 39, 2170–2177 (2006).

[b60] Casacuberta E. & Gonzalez J. The impact of transposable elements in environmental adaptation. Molecular Ecology 22, 1503–1517 (2013). 23293987 10.1111/mec.12170

[b61] Huang B. & Wu W. Mapping of Mutant Gene prbs Controlling Poly-Row-and-Branched Spike in Barley (Hordeum vulgare L.). Agric. Sci. China 10, 1501–1505 (2011).

[b62] Bai F. . Creating a New Wheat Strain of Early Maturity and Another One of Good Dwarf Quality by Introducing Exogeous Maize Nuclear DNA. Acta Agronomica Sinica. 25, 260–264 (1999).

[b63] Lu Y. & Zheng K. A simple method for isolation of rice DNA. Chinese Journal of Rice Science 6, 47–48 (1992).

[b64] Zhang Z., Schwartz S., Wagner L. & Miller W. A greedy algorithm for aligning DNA sequences. J. Comput. Biol. 7, 203–214 (2000). 10890397 10.1089/10665270050081478

[b65] Xu Z. & Wang H. LTR_FINDER: an efficient tool for the prediction of full-length LTR retrotransposons. Nucleic Acids Res. 35, 265–268 (2007). 10.1093/nar/gkm286 PMC1933203 17485477

[b66] Altschul S. F. . Gapped BLAST and PSI-BLAST: a new generation of protein database search programs. Nucleic Acids Res. 25, 3389–3402 (1997). 9254694 10.1093/nar/25.17.3389 PMC146917

