# Supplementary material for: Gene Deletion in Barley Mediated by LTR-retrotransposon BARE
Source: Sci Rep. 2017 Mar 2;7:43766. doi: 10.1038/srep43766 (PMC5333098; doi:10.1038/srep43766)
Supplement: Supplementary Information [file srep43766-s1.pdf]

Gene Deletion in Barley Mediated by LTR-retrotransposon *BARE*

Yi Shang[1#], Fei Yang[2,3#], Alan H. Schulman[4,5], Jinghuan Zhu[1], Yong Jia[3], Junmei Wang[1], Xiao-Qi Zhang[3], Qiaojun Jia[6], Wei Hua[1], Jianming Yang[1*], Chengdao Li[2,3*]

Supplementary Table 1. List of primers used for genetic mapping and identification of the deletion.

Supplementary Table 2. The Alignment Hit Table of each target sequence found by 15345-16193 as query hitting maize genome.

Supplementary Table 3. The Alignment Hit Table of each target sequence found by 16423-18000 as query hitting maize genome.

Supplementary Table 4. The Alignment Hit Table of each target sequence found by 18046-18087 as query hitting maize genome.

Supplementary Table 5. The Alignment Hit Table of each target sequence found by 27390-27543 as query hitting maize genome.

Supplementary Table 6. The Alignment Hit Table of each target sequence found by 25868-26648 as query hitting maize genome.

Supplementary Table 7. Genetic variations induced by introduction of exogenous genomic DNA.

Supplementary Figure 1. Linkage map of the *prbs* gene on chromosome 3H.

Supplementary Figure 2. *Vrs4* Haplotype analysis.

Supplementary Figure 3. Hits diagram of 15345-16193 on maize chromosomes.

Supplementary Figure 4. Hits diagram of 16423-18000 on maize chromosomes.

Supplementary Figure 5. Hits diagram of 18046-18087 on maize chromosomes.

Supplementary Figure 6. Hits diagram of 27390-27543on maize chromosomes.

Supplementary Figure 7. Hits diagram of 25868-26648 on maize chromosomes.

Supplementary Table 1. List of primers used for genetic mapping and identification of the deletion.

Primer Name	Forward sequence (5'-3')	Reverse sequence (5'-3')	Tm (˚C)	size (bp)
Cbic43	ATCCAAAGCTCCCAAATATC	TTTCTTCCGAATGCTAACTA	55	115
Cbic44	GATACAGCTATTTCCCCCAT	GGTTAGTCATTTTCCCGAGA	55	174
Cbic45	AGGTATTTGTCCCGCTTCATAG	CAGAGTAATCATGCACACTTGC	55	799
Cbic46	CATTCGGTATGTCGTTTTGTCC	GGAGTGGAGGGGAGATATAGAG	55	639
Cbic47	GATTAATCGATCCGTTTCGCAG	GAATAGTGCCGAGAATACTGCT	55	412
Cbic48	CGACACAGTACAAACGAATGTC	CACATCAGCTCACCAAGTAAGA	55	589
Cbic49	GGCATAACATGCATGGGTTTAG	GGATGAAGATTGGGGAAAGTGA	55	656
Cbic50	CCTAGAAAACAACAAGGGGGAT	ATCAATACTACTAGCAACCGCC	55	274
Cbic76	GAATGGTAGGGGCGATGGAG	CTCCATGCATCGTCCAGGTT	60	766
Cbic77	GTCAGCTAACGGTCAAAGGC	TCCAGCAGTTATGCCCAAGG	60	949
Cbic82	ACACATGCCCAATCAAGAGC	CGAACAATTCCACGCGATGA	60	898
Cbic83	CCGGTGCTAAGGTGTTGTTC	ATCTTGGGGCAGGCTTCTAG	60	775
Cbic85	TGAGGGAGGACCAATCTTCA	ACGGGTGATTTGGTGATGTT	58	1185
Cbic86	AGAATCGTCACTAAACGCCC	TGCTACTCAGATTCGTTGCC	58	581
Cbic87	AGATGGAGGCGATGAAGGTA	AGGTTATAACGGACGTTGCC	58	308
Cbic93	TCATTCGTCATGCACACACA	GGCTGTATAGTTCTCGCTG	58	1663
Cbic94	AACCAATGCAACTAGGGAGC	CTGGGCTGTATAGTTCTCGC	58	1099
Cbic95	ATCGGTTGCGAGTTTCCC	CCGGTTTACCAATCGGGAC	58	906
Cbic110	AGGAGGGTGTGTGGATGAAG	GCATTCCTTCGGTGCTAGTG	60	740
Cbic111	TCCACGTGCAACTCAGATCT	CCATCCAATCACAACCTGGC	60	654
Cbic112	TACAGAAAACCCCAGCCCAT	CTCACTTTTCCCCACTCCCT	60	702
Cbic113	TTGTTCTCGTGGACATGGGA	CCAACGACGAGCAAAAGGAA	60	766
Cbic116	AGTCCCTAGCTACTTCCCCA	AGAGAGATATCCCACGTGCG	60	608
Cbic117	CGGGAATGACGAATGGGTTC	TGAGCAACGGACTTACTCGT	60	968
Cbic118	CTGCCGCCAAGCTATAAAGG	ACGCTCCATTGTTCCTGAGA	60	966
Cbic119	TCGACAGTCACACCCTGTTG	CCATGTCTTCCTATGTGTCCGA	60	707
Cbic120	AGTAAGACTCAGTGCCCAGC	TAGTCGCGGATGAGGTTGAA	60	596
Cbic121	ATGCACCCAATGTGAGAGGA	TTTTCCGCTTTGGGTTCCAG	60	850
Cbic122	TAAAGATGACTGGCGGGTGT	ACCTTAGGATGCTACCGTCG	60	602
Cbic123	CCGGGGCAATAATAGGGTCT	TGCACACAAGGCATTTCTCC	60	890
Cbic131	CAAGTCTCTAATGTCCTCCATA GTCACC	CGACACCTCTCTCTCAAATTA TGTCGTC	68	42919
Cbic132	CGTCTTCAGAGGGAAAGATCTA ACATGGTG	AATCCCATGTCTTCCTATGTGT CCGATG	68	42820

Supplementary Table 2. The Alignment Hit Table of each target sequence found by 15345-16193 as query hitting maize genome.

Target ID	definition	e-value	percent identity	#
chr5	chr5	1.484e-75	73.01	99
chr9	chr9	6.905e-74	72.87	69
chr7	chr7	6.905e-74	72.87	74
chr6	chr6	6.905e-74	72.90	79
chr3	chr3	6.905e-74	72.90	76
chr1	chr1	6.905e-74	72.87	137
chr8	chr8	3.213e-72	72.78	92
chr4	chr4	3.213e-72	72.78	96
chr2	chr2	3.213e-72	72.75	133
chr10	chr10	3.213e-72	72.72	69
scaffold_495	scaffold_495 dna:scaffold scaffold:AGPv3:scaffold_495:1:380467:1	1.505e-65	72.38	1
scaffold_509	scaffold_509 dna:scaffold scaffold:AGPv3:scaffold_509:1:487110:1	3.282e-57	71.92	1

Supplementary Table 3. The Alignment Hit Table of each target sequence found by 16423-18000 as query hitting maize genome.

Target ID	definition	e-value	percent identity	#
chr3	chr3	2.035e-121	72.61	90
chr8	chr8	9.467e-120	72.52	106
chr4	chr4	9.467e-120	72.52	93
chr7	chr7	4.405e-118	72.45	89
chr6	chr6	4.405e-118	72.47	93
chr5	chr5	4.405e-118	72.47	110
chr2	chr2	4.405e-118	72.49	134
chr10	chr10	4.405e-118	72.45	77
chr9	chr9	2.049e-116	72.38	79
chr1	chr1	2.049e-116	72.38	146
scaffold_495	scaffold_495 dna:scaffold scaffold:AGPv3:scaffold_495:1:380467:1	9.602e-110	72.26	1
scaffold_509	scaffold_509 dna:scaffold scaffold:AGPv3:scaffold_509:1:487110:1	4.467e-108	75.59	1

Supplementary Table 4. The Alignment Hit Table of each target sequence found by 18046-18087 as query hitting maize genome.

Target ID	definition	e-value	percent identity	#
chr2	chr2	3.378e-12	97.62	122
chr1	chr1	3.378e-12	97.62	128
scaffold_509	scaffold_509 dna:scaffold scaffold:AGPv3:scaffold_509:1:487110:1	1.571e-10	95.24	1
scaffold_495	scaffold_495 dna:scaffold scaffold:AGPv3:scaffold_495:1:380467:1	1.571e-10	95.24	1
chr9	chr9	1.571e-10	95.24	65
chr8	chr8	1.571e-10	95.24	84
chr7	chr7	1.571e-10	95.24	74
chr6	chr6	1.571e-10	95.24	77
chr5	chr5	1.571e-10	95.24	88
chr4	chr4	1.571e-10	95.24	84
chr3	chr3	1.571e-10	95.24	79
chr10	chr10	1.571e-10	95.24	76

Supplementary Table 5. The Alignment Hit Table of each target sequence found by 27390-27543 as query hitting maize genome.

Target ID	definition	e-value	percent identity	#
chr3	chr3	3.106e-45	88.20	1

Supplementary Table 6. The Alignment Hit Table of each target sequence found by 25868-26648as query hitting maize genome.

Target ID	definition	e-value	percent identity	#
chr3	chr3	5.543e-169	81.10	3
chr8	chr8	6.569e-49	80.86	5
chr9	chr9	8.618e-38	78.14	4
chr5	chr5	8.618e-38	78.29	3
chr1	chr1	1.115e-36	78.12	9
chr6	chr6	1.452e-30	75.69	5
chr4	chr4	4.096e-21	74.65	3
chr10	chr10	1.919e-14	79.69	2
chr2	chr2	5.412e-5	85.19	1

Supplementary Table 7. Genetic variations induced by introduction of exogenous genomic DNA.

DNA donor	Recipient	Method	Trait	Reference
Sorghum	Wheat	Pollen tube	Glutenin, tolerance	Zhou et al. 1992[1], Pei et al. 1999[2], Wang et al. 2000[3], Ni et al. 2005a[4], Ni et al. 2005b[5].
Sorghum	Wheat	Pollen tube	Stripe-rust resistance, quality indicators	Wang et al. 1995[6], Ou et al. 2013[7]
Barley	Wheat	Pollen tube	Stable immunity and high-resistance to powdery mildew	Yan et al. 1994a[8], Yan et al. 1994b[9].
Wheat (white seed)	Wheat (red seed)	Pollen tube	Seed colour	Liu et al. 1994[10].
Wheat (Fuzhuang 30 and Xiaobaidongmai)	Wheat (beinong 6)	Pollen tube	High-resistance to powdery mildew	Wang et al. 2003[11].
Elytrigia elongata	Wheat	Pollen tube	Rust resistance	Wang et al. 1995[6], Qin et al. 1997[12].
Elytrigia elongata	Wheat	Pollen tube	Stripe rust resistance	Ou et al. 2005[13].
Reed	Wheat	Pollen tube	The awn property, grain color, plant height, ear length, grain number of each ear and sharp or dull of grain husk point,	Liu et al. 1998[14].

			especially salt resistance	
Maize	Wheat	embryo soaking	Plant height, protein content , lysine content, earlier matured,	Bai et al. 1999[15].
Maize	Wheat	Pollen tube	Length and square of the flag leaf, ear shape and disease resistance	Hu et al. 2007[16].
Leymus racemosus	Wheat	Pollen tube	Exogenous DNA fragment	Miao et al. 2000[17], Zhang et al. 2000[18].
Agropyron elongatum	Wheat	Pollen tube	Plant height, plant type, spike type, disease resistance and stress tolerance, lower saline damage index, higher saline tolerance, high ratio of SOD activity of flag leaves to the top second leaves, high saline and drought tolerance	Huang et al. 2000[19].
Soybean	Wheat	Pollen tube	Protein content and amino acid content, gene of wild soybean	Wang et al. 2003[20].
Oat (*Avena sativa* L.)	Wheat	Pollen tube	Flag leaf area, spike length, grain number of main spike, grain weight of main spike and weight of thousand grains	Liu et al. 2006[21].
T. Aegilopoides Bal, *T. Dicoccoides* korn, T. *Timopheevi* zhuk, and *T. Polonicum* L.	Wheat	injection to ovule or transduction through pollen tube path	Protein content, auricles color etc.	Kong et al. 1992[22].
Spartina angelica	Rice	injection to ovule	Plant shape with broad, short, stiff and erect leaves, contents of the	Duan et al. 1985[23].

			protein and 16 amino acids	
Maize	Rice	imbibing rice seed with corn DNA solution.	Amylase content, the percentage of albumin, globulin and glutelin in the seeds	Chen et al. 1997[24].
Sorghum	Rice	Pollen tube	Net photosynthesis rates, exogenous DNA fragment	Hong et al. 1999[25], Lou et al. 2004[26], Lou et al. 2005[27].
Sorghum	Rice	spike-stalk injection	Exogenous DNA fragment	Zhao et al. 2001[28], Zhao et al. 2004[29]
Wild rice	Rice	Pollen tube and stem injecting	Exogenous DNA fragment	Li et al. 2006[30].
Wild rice	Rice	spike-stalk injection	Plant type, grain quality and grain yield, exogenous DNA, specific SNPs and InDels.	Zhao et al. 2001[28], Wang et al. 2006[31]. Sun et al. 2013[32].
Yunnan *O. Officinalis* and oats	Rice	pollen tube or by injecting exogenous DNA into the stem of rice in boot stage	Growth period, shape of plant, content of protein and amino acid, resistance to rice blast and isoenzymes of esterase and peroxidase, rice blast resistance	Song et al. 1993[33].
Kidney bean	Maize	pollen grain introduction	Progenies have characteristic type of donor, and most of traits can stable in D4 generation	Qi et al. 1995[34].
Soybean	Maize	Pollen tube	High-protein, plant pattern and anther color	Qi. 2000[35] Ding et al. 2001[36].
Tripsacum and	Maize	Pollen tube	High combining ability	Yang et al.

Teosinte			of yield	2005[37].
Sugarcane	Maize	seed-immersion	The height of plant, the height of fruit, the number of leaf, the color and morphology of leaf, the length and width of leaf, the area of leaf, the weight of 1000 grain and the period of growth, photosynthetic pigment contents and quality	Zhang et al.2006[38], Zhang et al. 2008[39].
Waxy corn	Sweet maize	Pollen tube	Height of plant, the height of fruit, the area of leaf, sugar, protein and starch content	Zhang et al. 2006[40].
Island Cotton (*Gossypium barbadense* L.)	Upland Cotton (*Gossypium hirsutum* L.)	Injection donor DNA into ovaries	Morphological characters, growing period, vegetative organs, reproductive organs and other characters of economic importance	Huang et al. 1981[41].
Island cotton	Upland cotton	Pollen tube	Highly resistance to *Fusarium* wilt	Zhou et al. 1983[42].
Island cotton	Upland cotton	Pollen tube	High quality of fiber, morphology, exogenous DNA fragments	Wang et al. 2005[43], Wang et al. 2006[44].
Fusarium wilt-resistant cotton cultivar	Susceptible cotton cultivars	Injection donor DNA into ovaries	Disease resistance and yields	Huang et al. 1986[45].
Wild soybean	Cultivated soybean	Pollen tube	Protein content, globin content, fat content, yield	Lei et al. 1995[46], Lei et al. 2000[47].
Soybean	Soybean	Pollen tube	Exogenous DNA fragments (RAPD)	Lei et al. 1996[48].
Soybean landrace, wild soybean and	Cultivated soybean	Pollen tube	Yield, SMV resistance	Liu et al. 1997[49].

Gleditsia japonica				
Cotton	Ramie	soaking embryo	Exogenous DNA fragments (RAPD)	Zhang et al. 2000[50].
Squash	Watermelon	Injection donor DNA into ovaries	Wilt resistance	Chen et al. 1998[51].
Pumpkin	Watermelon	embryo injection	Resistance to *Fusarium oxysporum* sp. niveum, new special rind color and strip	Wang et al. 2002[52].
Pineapple	Cucumber	Pollen tube	Yield, the content of sugar	Chen et al. 1998[3]. Chen et al. 2002[54]
Rhizophora apoculata	*Capsicum annuum*	Pollen tube	Salt tolerance	Lin et al. 1999[55].
Rhizophora apoculata	*Solanum melongena*	Pollen tube	Salt tolerance, growth, rate of transpiration and photosynthesis, the activity of pepcase and the morphology of the blade stomas.	Lin et al. 2001[56].
Rhizophora apoculata	Alfalfa (*Medicago sativa*)	Pollen tube	Salt tolerance, exogenous DNA fragments (RAPD)	Zhang et al. 2011[57].
Suaeda salsa	Tomato	Pollen tube	Salt tolerance	Xiao et al. 2008[58].

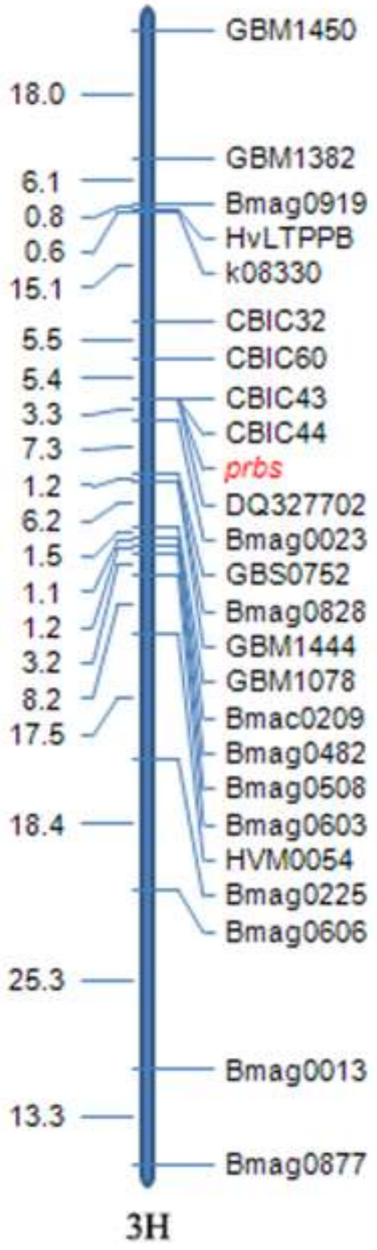

Supplementary Figure 1. Linkage map of the *prbs* gene on chromosome 3H.

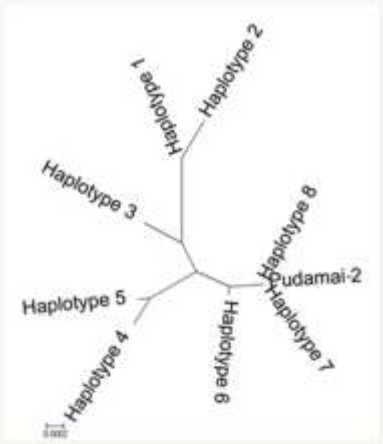

sequence name	SNP site																			
	96	171	244	840	841	842	843	844	845	846	847	848	849	941	971	972	1089	1128	1475	1554
Haplotype 1	T	C	C	T	T	C	C	C	A	G	A	A	C	A	-	-	G	C	G	A
Haplotype 2	.	.	.	.	.	.	.	.	.	.	.	.	.	.	-	-	.	.	A	.
Haplotype 3	.	.	.	.	.	.	.	.	.	.	.	.	.	.	-	-	A	T	.	G
Haplotype 4	-	T	T	.	.	.	.	.	.	.	.	.	.	G	-	-	.	.	.	.
Haplotype 5	-	T	T	.	.	.	.	.	.	.	.	.	.	G	-	-	.	.	.	G
Haplotype 6	-	T	T	-	-	-	-	-	-	-	-	-	-	.	-	-	.	T	.	G
Haplotype 7	-	T	T	-	-	-	-	-	-	-	-	-	-	.	-	-	A	T	.	G
Haplotype 8	-	T	T	-	-	-	-	-	-	-	-	-	-	.	T	A	A	T	.	G
Pudamai-2	-	T	T	-	-	-	-	-	-	-	-	-	-	.	T	A	A	T	.	G

Supplementary Figure 2. *Vrs4* Haplotype analysis.

The primer pairs specific to *Vrs4* with overlapping fragments covering 2046 bp (Koppolu R et al. 2013) were amplified for direct PCR sequencing from wild barley cultivars Pudamai-2. Haplotypes of *Vrs4* were from GenBank (accession nos: KC854546 to KC854553) (Koppolu R et al. 2013). A phylogenetic tree was constructed using the MEGA6 program (Tamura K et al. 2013) with the minimum evolution method. The result showed that haplotype of *Vrs4* from Pudamai-2 was same as haplotype 8, which was characteristic of a inset of 'TA' bases in 5' UTR of *Vrs4*.

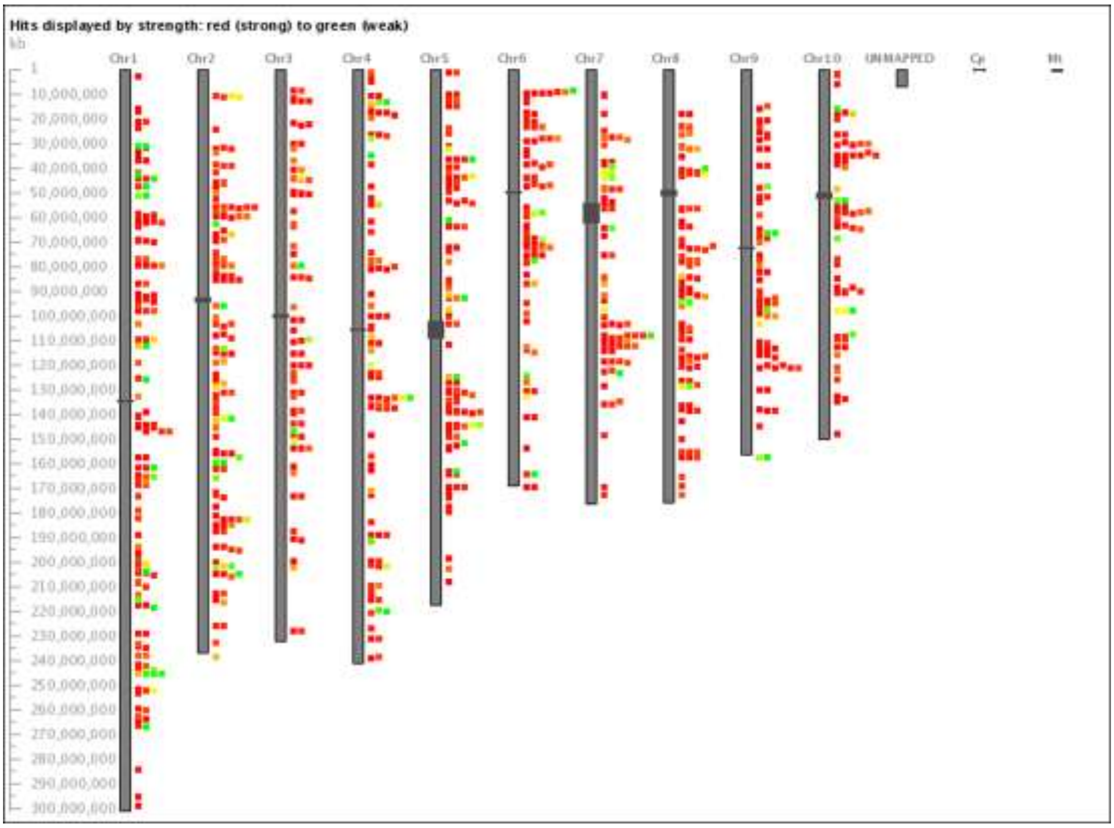

Supplementary Figure 3. Hits diagram of 15345-16193 on maize chromosomes.

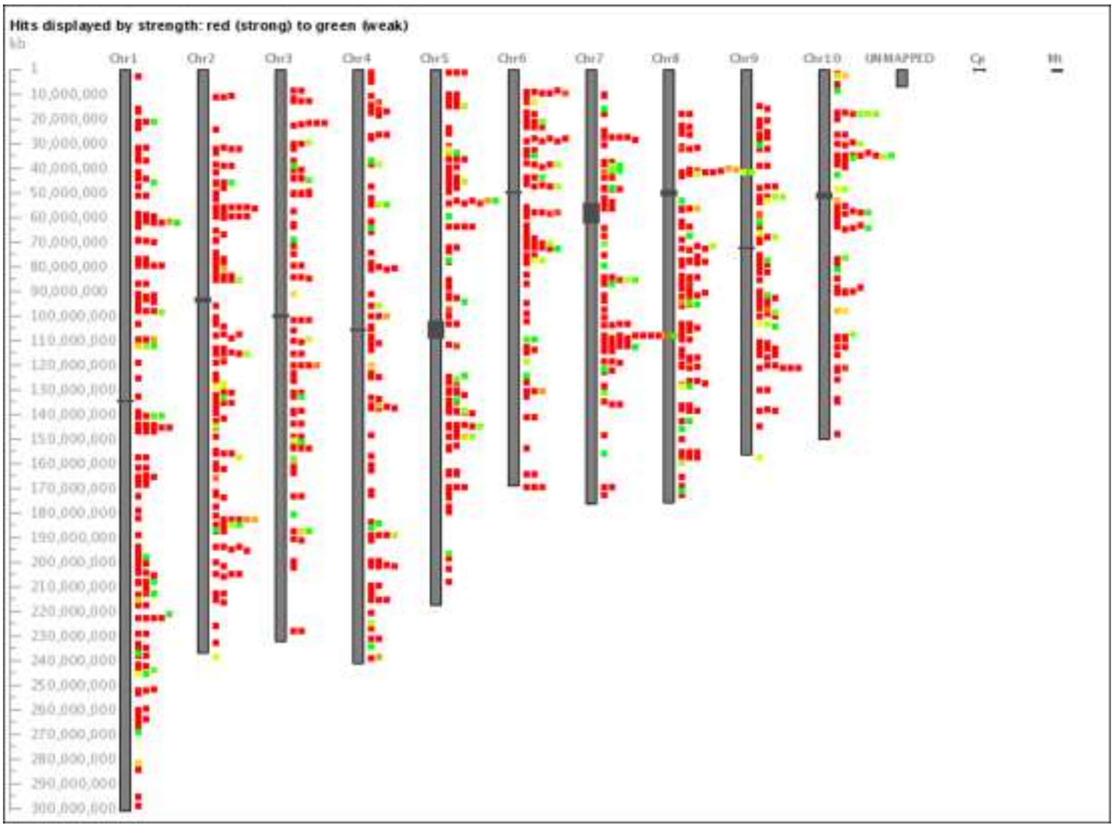

Supplementary Figure 4. Hits diagram of 16423-18000 on maize chromosomes.

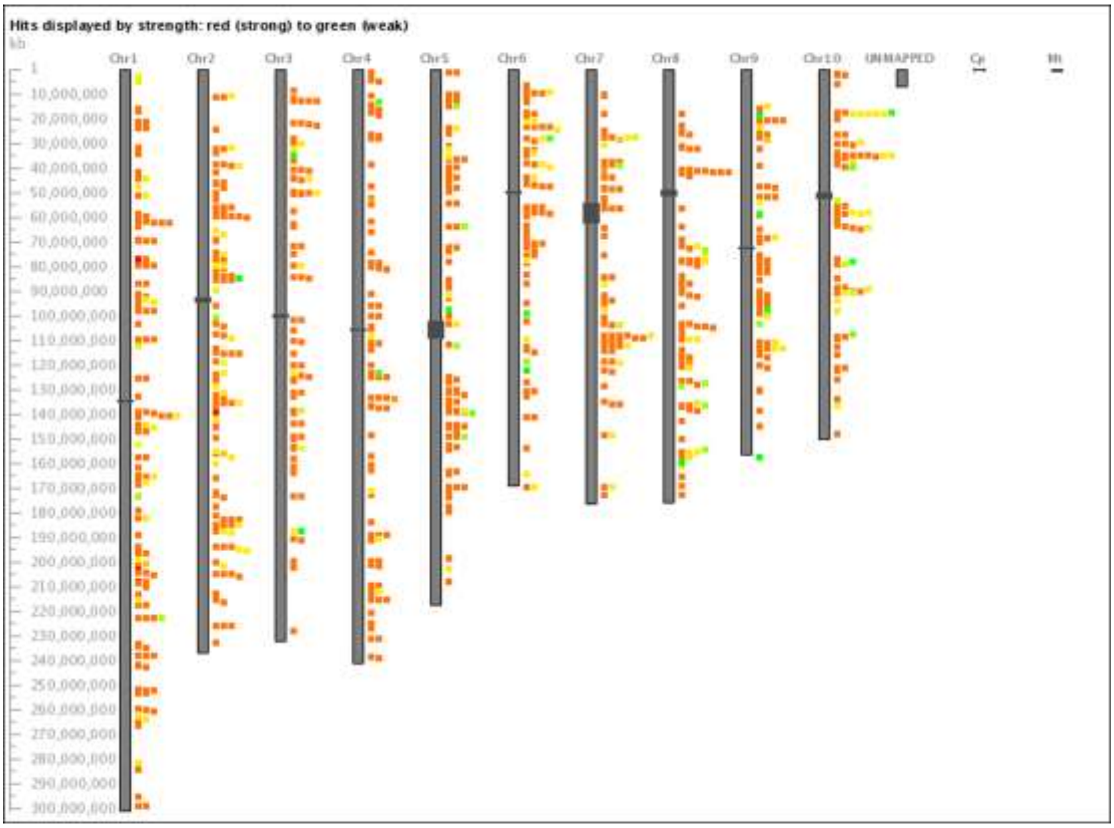

Supplementary Figure 5. Hits diagram of 18046-18087 on maize chromosomes.

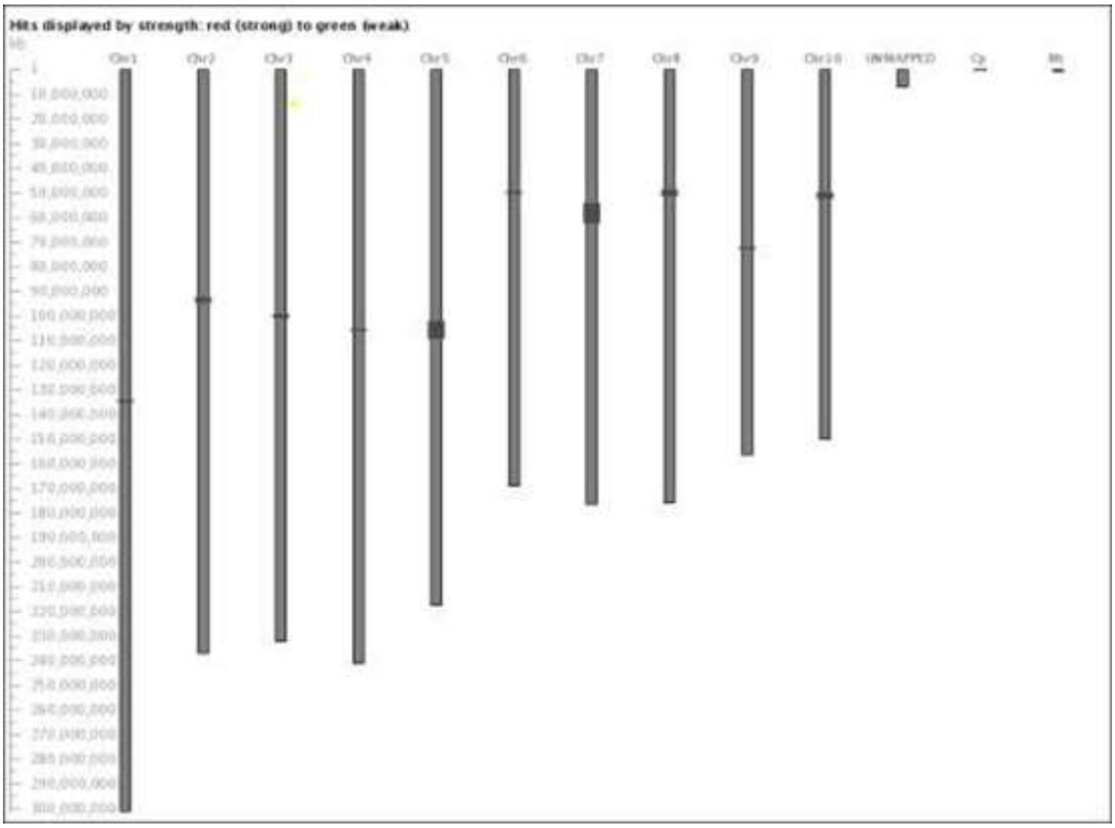

Supplementary Figure 6. Hits diagram of 27390-27543on maize chromosomes.

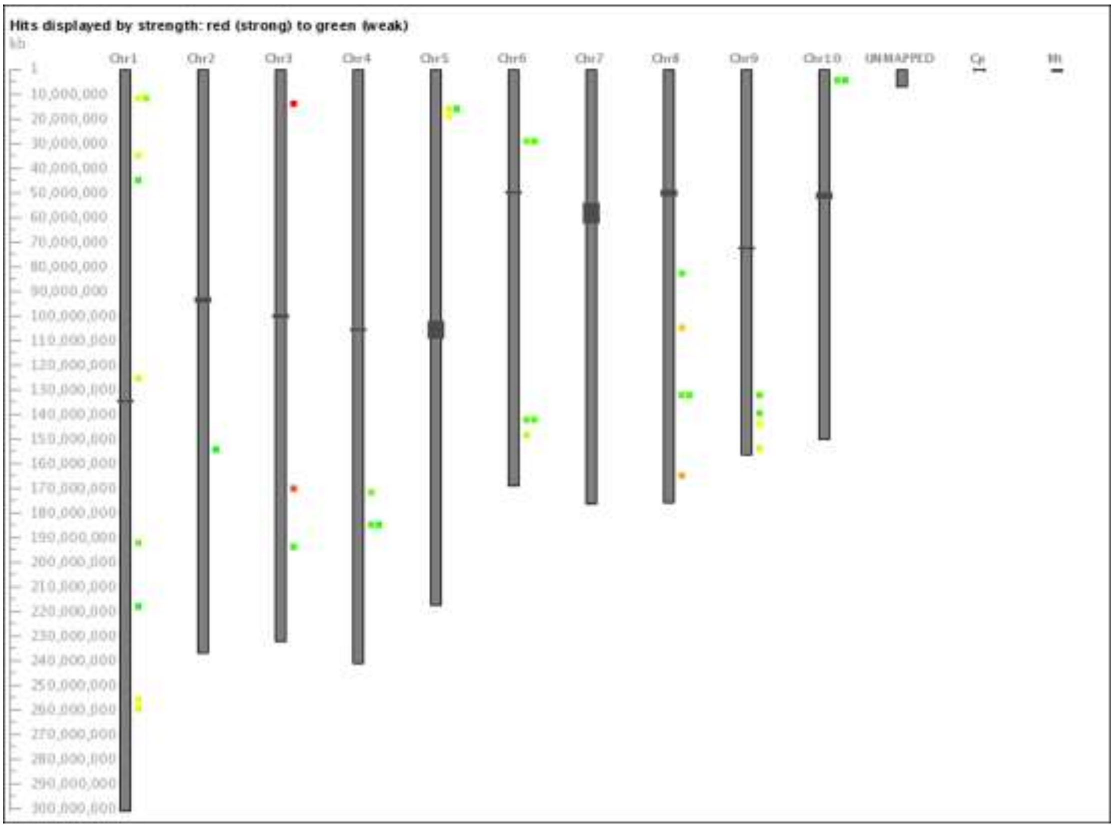

Supplementary Figure 7. Hits diagram of 25868-26648 on maize chromosomes.

References:

1. Zhou, W. L. et al. Studies on DNA transfer from C4 crop to wheat. *Acta Agronomica Sinica*. **18**, 418-423 (1992).
2. Pei, X. W. et al. Selection and RAPD identification of a new wheat line with high yield and stress resistance by introducing sorghum DNA. *Journal of Lanzhou University*. **35**, 130-135 (1999)
3. Wang, X. J., Li, X. L., Wang, Y. F. The changes of the HMW glutenin subunits of introducing sorghum DNA into spring wheat. *Acta botanica boreali-occidentalia sinica*. 20, 979- 983 (2000).
4. Ni, J. F., Ling, L. J., Ou, Q. M., Ye, C. L., Wu, L. G. Molecular breeding by introducing exogenous DNA into wheat. *Journal of Triticeae Crops*. **25**, 27-31 (2005a).
5. Ni, J. F. et al. The change of the HMW glutenin subunits by introducing sorghum DNA into spring wheat. *Journal of Lanzhou University*. **41**, 47-49 (2005b).
6. Wang, Y. F., Chen, K. M., Jiao, C. J., Zhou, W. L., Ni, J. F. Studies on biological characters and endosperm protein of variation lines obtained by introducing exogenous DNA into wheat. *Acta Agronomica Sinica*. **21**, 404-441 (1995).
7. Ou, Q. M. et al. Molecule -pyramiding breeding of new improved wheat varieties by introducing sorghum DNA via pollen-tube pathway. *Agricultural Research in the Arid Areas*, **31**, 6-12 (2013).
8. Yan, X. F., Liu, W. X., Wang, S. J., Wang, X. C., Wang, X. F. Inheritance of powdery mildew resistance in variants obtained by introducing barley DNA into wheat. *Hereditas (Beijing)*. **16**, 26-30 (1994a).
9. Yan, X. F., Liu, W. X., Lin, Q. P. A study on the esterase isoenzyme of the variants derived from barley DNA introduction into common wheat. *Acta Agricultuaae Boreall-Sinica*. **9**, 39-43 (1994b).
10. Liu, G. Q. et al. Direct transformation of foreign DNA uptake during wheat pollinating and its application in crop breeding. *Acta Genetica Sinica*. **21**, 463-467 (1994).
11. Wang, L. X. et al. Transgenic wheat lines by transferring powdery mildew resistance gene through pollen tube pathway. *Scientia Agricultura Sinica*. **36**, 1576-1581 (2003).
12. Qin, L. et al. Rapid verification of rust resistant wheat variants. *Acta botanica boreali-occidentalia sinica*. **17**, 22-26 (1997).

13. Ou, Q. M., Ni, J. F., Zhang, Z. Y., Chen, Y. L. Variation in winter wheat characters arising from incorporation of E.elongata DNA and their genetic analysis. *Acta Tritical Crops*. **25**, 18-22 (2005)
14. Liu, Z. S., Zhang, K. P., Fu, X. Y. Reed DNA introducing into wheat and salt resistant transformation screening. *Jilin Agricultural Sciences*. **4**, 29-31 (1998).
15. Bai, F. et al. Creating a New Wheat Strain of Early Maturity and Another One of Good Dwarf Quality by Introducing Exogeous Maize Nuclear DNA. *Acta Agronomica Sinica*. **25**, 260-264 (1999).
16. Hu, W. M., Xu, C. L., Wang, Y. G. Characteristics of the transgenic wheat D1 generation from total corn DNA through pollen tube pathway. *Hubei Agricultural Sciences*. **46**, 867-869 (2007).
17. Miao, J., Zhao, M. A., Li, W. Q. Molecular proof of wheat transformed by total DNA of Leymus racemosus. *Acta genetica sinica*. **27**, 621-627 (2000).
18. Zhang, M. Y. et al. Introduction of Leypus racemosus Lam Tzvel DNAs into common wheat through pollen-tube pathway. *Journal of Agricultural Biotechnology*. **8**, 165-168 (2000).
19. Huang, C. Y., Chu, X. S., Yang, P. P. Using extraneous DNA introduction method to breed drought and saline tolerant wheat variety. *Shandong Agricultural Sciences*. **(4)**, 4-6 (2000).
20. Wang, X. L. et al. Characterization of wheat (Triticum Aestivum L) introduced wild soybean DNA and molecular identify by RAPD. *Journal of Nankai University*. **36**, 37-40 (2003).
21. Liu, P., Kang, Z. S. Oat (Avena sat iva L) exogenous DNA introduction into common wheat and PAPD analysis. *Agricultural Research in the Arid Areas*. **24**, 100-103 (2006).
22. Kong, Q., Xu, N. Y., Lin, Q. A study on the introduction of exogenous DNA and variation of wheat. *Journal of Wuhan Botanical Research*. **10**, 339-344 (1992).
23. Duan, X. L., Chen, S. B. Variation of the characters in rice (Oryza Sativa). *Scientia Agricultura Sinica*. **33**, 104-106 (1985).
24. Chen, X. B., Liao, A. J., Luo, Z. M. The seed component variations induced by the introduction of exogenous DNA into rice. *Journal of Hunan Agricultural University*. **23**, 19-22 (1997).
25. Hong, Y. H., Dong, Y. Y., Zhao, Y., Xiao, L. T. Studies on the introduction of sorghum total DNA into rice. *Journal of Hunan Agricultural University*. **25**,

87-91 (1999).
26. Luo, H. F., Zhong, B. Q., Yang, Z. L., Li, Y. F., He, G. H. The SSR molecular evidence of rice transformation via pollen tube pathway. *Molecular Plant Breeding*. **2**, 501-505 (2004).
27. Luo, H. F., Yang, Z. L., Zhong, B. Q., Li, Y. F., He, G. H. 2005. The SSR molecular analysis of rice transformation via pollen tube pathway. *Chinese Agricultural Science Bulletin*. **21**, 28-30 (2005).
28. Zhao, B. R., Jia, J. H., Wang, Q., Wang, B., Yuan, L. P. Comparison of the nucleotide sequence of the specific DNA fragments between the variant line and its donor. *Hybrid Rice*. **16**, 46-49 (2001).
29. Zhao, B. R. et al. SSR analysis of the variants from rice maintainer lines transferred exogenous DNA. *Hybrid Rice*. **19**, 47-50 (2004).
30. Li, S. H., Yang, Q. W., He, J., Han, G. M. SSR detection of rice progenies with transformed Oryza rufipogon DNA. *Acta Bot Boreal Occident Sin*. **26**(4), 672-676 (2006).
31. Wang, S. W. et al. Molecular verification of DNA flow from wild rice (O. minuta) to cultivated rice. *Scientia Agricultura Sinica*. 39, 2170-2177 (2006).
32. Sun, Y. D. et al. Phenotypic difference and gene variation in agronomic traits between a rice variant with introduced exogenous genomic DNA and its receptor. *Hybrid Rice*. **28**, 62-67 (2013).
33. Song, L. R. et al. Variant rice plant obtained by introducing exogenous DNA into paddy rice ‘Southwest 175’. *Southwest China Journal of Agricultural Sciences*. **6**, 21-25 (1993).
34. Qi, Y. H. et al. A preliminary study on the introduction transformation and variation of character of maize inbred lines of kidney bean DNA. *Biotechnology*. **5**, 29-32 (1995).
35. Qi, Y. H. Study on introducing soybean DNA directly into inbred line maize. *Maize Sciences*. **8**, 34- 36 (2000).
36. Ding, M. Z. et al. A study on the screening high-protein variants by transmitting exogenous soybean DNAs into maize. *Southwest China Journal of Agricultural Sciences*. **14**, 8-12 (2001).
37. Yang, L. G., Wang, J. Y., Shi, T. Y., Wang, Y., Jiang, W. C. A novel method to breed new maize inbred lines and their hybrids by transformation of exogenous DNA. *Rain Fed Crops*. **25**, 283-285 (2005).
38. Zhang, H. Y., Lu, P., Yu, W. H., Hao, X. Q., Wang, Y. Study on the mutant

character of waxy maize inbred line offsprings introducing sugarcane DNA. *Journal of Maize Sciences*. **14**, 32-34 (2006).

39. Zhang, H. Y., Liu, A. H., Xue, Y. X. Study on traits variation of inbred line of corn inducted from sugarcane DNA. *Hubei Agricultural Sciences*. **47**, 498- 500 (2008)
40. Zhang, H. Y., Feng, R., Lu, P., Yu, W. H., Hao. X. Q. Study on the mutant character of sweet maize inbred line offsprings by transmitting exogenous DNA. *Chinese Agricultural Science Bulletin*. **22**, 186-188 (2006).
41. Huang, J. Q. et al. Variations in the characters of Upland cotton (Gossypium hirsutum) induced by exotic DNA of Sea-island Cotton (Gossypium barbadense). *Acta Genetica Sinica*. **8**, 56-62 (1981).
42. Zhou, G. Y. et al. Introduction of exogenous DNA into cotton embryos. *Methods Enzymol*. **101**, 433-481 (1983).
43. Wang, F. R. et al. Genetic analysis of upland cotton germplasm obtained from introduced DNA from island cotton. *Scientia Agricultura Sinica*. **38**, 1528-1533 (2005).
44. Wang, F. R. et al. Variant germplasm obtained by pollen tube pathway technique and SSR analysis. *Shandong Agricultural Sciences*. **(1)**, 10-12 (2006).
45. Huang, J. Q. et al. Transfer of Fusarium wilt resistance between cotton cutivars via exogenous DNA introduction. *Scientia Agricultura Sinica*. **(3)**, 32-36 (1986).
46. Lei, B. J. et al. New soybean strains of high protein and double high content obtained from introduction of exogenous total DNA. *Soybean Science*. **14**, 203-207 (1995).
47. Lei, B. J. et al. Breeding of a high-yielding, high quality and high-protein content soybean cultivar heisheng 101 through direct introduction of alien DNA. *Acta Agronomica Sinica*. **26**, 725-730 (2000).
48. Lei, B. J. et al. Early maturing lines of obtained through foreign total DNA introduction in soybean. *Acta Agronomica Sinica*. **22**, 173-177 (1996).
49. Liu, D. P., Liao, L., Yuan, Y., Liu, Y. Z. Gaining of soybean lines resistant to SMV by introducing exogenous DNA. *Soybean Science*. 16, 277-282 (1997).
50. Zhang, F. Q., Jiang, J. X., Li, Z. D., Zheng, S. X., Zhou, G. Y. Studies on variation of introducing cotton DNA into ramie. *Scientia Agricultura Sinica*. **33**, 104-106 (2000).

51. Chen, W. S. et al. Gene transfer via pollen-tube pathway for anti-fusarium wilt in watermelon. *Biochem Mol Biol Int*. **46**, 1201-1209 (1998).
52. Wang, H. B. et al. Study on the selection of new watermelon germplasm for resistance to fusarium wilt disease by introducing pumpkin DNA. *Acta Agriculturae Boreali-occidentalis Sinica*. **11**, 24-27 (2002).
53. Chen, X. H., He, H. X., Zeng, H. Preliminary studies on the introduction of pineapple DNA into cucumber. *Natural science journal of Hainan university*. **16**, 62-68 (1998).
54. Zhao, B. R. et al. RAPD analysis of new rice strains developed through the method of spike stalk injecting DNA from wild relative. *Acta Agronomica Sinica*. **15**, 424-430 (2000).
55. Lin, Q. F., Deng, Y. C., Wu, D. G., Chen, J. P., Li, G. Y. Molecular breeding of salt-resistant Capsicum annuum. *Progress in Biotechnology*. **19**, 19-24 (1999).
56. Lin, Q. F., Deng, Y. C., Huang, W., Chen, J. P., Li, G. Y. Introducing total DNA of Rhizothora apicularta to Solanum melongena to generate salt-tolerant progenies. *Progress in Biotechnology*. **21**, 40-44 (2001).
57. Zhang, L. Q., Niu, Y. D., Hao, J. F., Hasi, A. 2011. Production and RAPD verification of T0 salt-tolerant alfalfa introducing the total DNA from Rhizophora apiculata by the pollen-tube pathway. *Acta Prataculturae Sinica*. **20**, 292-297 (2011).
58. Xiao, J., Zhang, L. J., Ren, Z. Y., Chen, X., Zha,o Y. To create new salt resistance tomato line using the pollen tube pathway. *Journal of Shenyang Agricultural University*. **39**, 362- 364 (2008).
